# Peritumoral Delivery of Docetaxel‐TIPS Microparticles for Prostate Cancer Adjuvant Therapy

**DOI:** 10.1002/adtp.202000179

**Published:** 2020-10-19

**Authors:** Ketevan Paliashvili, Alexander Popov, Tammy L. Kalber, P. Stephen Patrick, Angela Hayes, Alan Henley, Florence I. Raynaud, Hashim U. Ahmed, Richard M. Day

**Affiliations:** ^1^ Centre for Precision Healthcare UCL Division of Medicine University College London Gower Street London WC1E 6BT UK; ^2^ Centre for Advanced Biomedical Imaging UCL Division of Medicine University College London Gower Street London WC1E 6BT UK; ^3^ Drug Metabolism Pharmacokinetics and Metabolomics Cancer Research UK Cancer Therapeutics Unit at The Institute of Cancer Research Division of Cancer Therapeutics 15 Cotswold Road Sutton London SM2 5NG UK; ^4^ Division of Surgery Department of Surgery and Cancer Imperial College London South Kensington Campus London SW7 2AZ UK; ^5^ Centre for Precision Healthcare UCL Division of Medicine University College London Gower Street London WC1E 6BT UK

**Keywords:** adjuvant chemotherapy, docetaxel, microparticles, positive resection margin, prostate cancer, radical prostatectomy

## Abstract

Recurrence of prostate cancer after radical prostatectomy is a consequence of incomplete tumor resection. Systemic chemotherapy after surgery is associated with significant toxicity. Improved delivery methods for toxic drugs capable of targeting positive resection margins can reduce tumor recurrence and avoid their known toxicity. This study evaluates the effectiveness and toxicity of docetaxel (DTX) release from highly porous biodegradable microparticles intended for delivery into the tissue cavity created during radical prostatectomy to target residual tumor cells. The microparticles, composed of poly(dl‐lactide‐*co*‐glycolide) (PLGA), are processed using thermally induced phase separation (TIPS) and loaded with DTX via antisolvent precipitation. Sustained drug release and effective toxicity in vitro are observed against PC3 human prostate cells. Peritumoral injection in a PC3 xenograft tumor model results in tumor growth inhibition equivalent to that achieved with intravenous delivery of DTX. Unlike intravenous delivery of DTX, implantation of DTX‐TIPS microparticles is not accompanied by toxicity or elevated systemic levels of DTX in organ tissues or plasma. DTX‐TIPS microparticles provide localized and sustained release of nontoxic therapeutic amounts of DTX. This may offer novel therapeutic strategies for improving management of patients with clinically localized high‐risk disease requiring radical prostatectomy and other solid cancers at high risk of positive resection margins.

## Graphical abstract

Docetaxel (DTX) release from highly porous biodegradable microparticles is investigated for the treatment of prostate cancer. Tumor growth inhibition is equivalent to that achieved with intravenous delivery of DTX while avoiding the toxicity associated with intravenous delivery. DTX thermally induced phase separation microparticles offer a novel therapeutic strategy for solid cancers associated with the risk of positive resection margins.

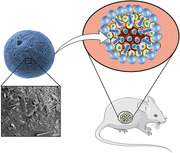

## 1 Introduction

Prostate cancer is the second most commonly diagnosed cancer in men and a leading cause of cancer related deaths.^[^ ^1^, ^2^ ^]^ Recent years have seen many patients benefitting from the availability of new therapeutics, advanced functional imaging, identification of biomarkers for prognosis and better use of existing therapies.^[^ ^3^ ^]^ For patients with localized prostate cancer, radical prostatectomy has remained a preferred treatment option, with laparoscopic and robot‐assisted procedures devised to reduce blood loss, retain continence, and improve recovery times compared with open surgery.^[^ ^4^, ^5^ ^]^ Although radical prostatectomy provides improved survival rates for localized prostate cancer,^[^ ^6^ ^]^ incomplete removal of the prostate occurs in ≈30% patients, resulting in biochemical recurrence and associated disease relapse with poor prognosis.^[^ ^7^, ^8^ ^]^ In the US, positive surgical margins following radical prostatectomy, indicative of incomplete cancer resection, has a reported incidence of ≈20%.^[^ ^9^ ^]^ The presence of a positive surgical margin requires adjuvant, salvage (radiotherapy) or hormonal therapy. Patients who fail these therapies require third line hormonal or chemotherapy drugs, such as DTX that confer higher risk of toxicity. Radiotherapy given after surgery to the prostate bed increases the risk of incontinence and erectile dysfunction as well as rectal problems such as bleeding, diarrhea and discomfort.

DTX is a taxane‐based chemotherapeutic that stops the growth of tumor cells by binding to intracellular β‐tublin, which disrupts microtubular function via assembly of microtubules resulting in mitotic arrest and ultimately cell death.^[^ ^10^ ^]^ Use of DTX as an adjuvant or neoadjuvant chemotherapeutic with radical prostatectomy in patients with clinically localized, high‐risk prostate cancer may improve recurrence‐free survival.^[^ ^11^, ^12^ ^]^ However, systemic administration of DTX is associated with a range of toxicity‐related side effects including neutropenia, leukopenia, neurological toxic effects, diarrhea, alopecia, asthenia, and nausea.^[^ ^13^ ^]^

Given the accessibility of residual cancer in the prostate bed or tissue cavity due to positive surgical margins following excision of the prostate, we have previously proposed the use of biodegradable microparticles to enable the targeted delivery of DTX as an adjuvant therapy during surgical removal of the prostate for cancer.^[^ ^14^ ^]^ Our previous study established an optimized process for loading TIPS microparticles with DTX and demonstrated in vitro their potential utility for tumor growth inhibition over a period of 12 days. The antisolvent precipitation method used for loading TIPS microparticles with DTX differs from other methods used in previous drug‐delivery studies with TIPS microparticles and results in drug being predominantly coated onto the microsphere surface.^[^ ^15^, ^16^, ^17^ ^]^ Following on from our preliminary studies, here we have evaluated the in vivo performance of DTX‐TIPS microparticles, including the pharmacokinetic profile, efficacy for inhibiting tumor growth and tolerability using a human prostate tumor xenograft model. The therapeutic index of DTX delivered via TIPS microparticles is shown to be improved compared with a conventional intravenous method of delivery, with inhibition of tumor growth achieved in the absence of systemic toxicity. TIPS microparticle delivery of DTX, together with the facile formulation method for coating the microparticles with drug, provides a promising strategy for effectively managing the risk of positive surgical margins associated with radical prostatectomy in prostate cancer and other localized, surgically resectable solid cancers.

## 2 Results and Discussion

### 2.1 Immobilization of DTX onto PLGA TIPS Microparticles

**Figure 1:**
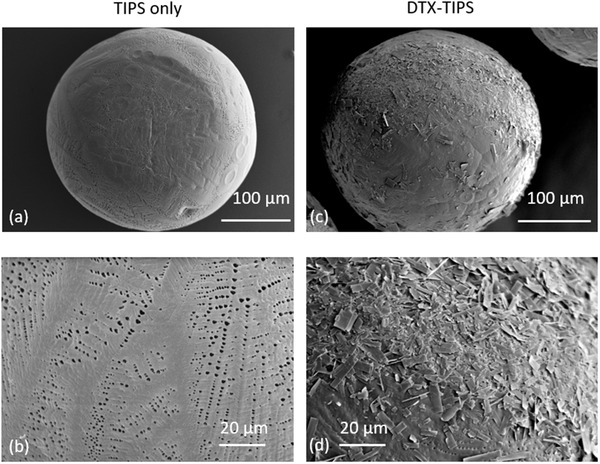
Ultrastructural imaging of 10% (w/v) PLGA TIPS microparticles. a) Unloaded control PLGA TIPS microparticle. b) Higher magnification of unloaded control PLGA TIPS microparticle with the porous surface features of the microparticles visible. c) PLGA DTX‐ TIPS microparticle. d) Higher magnification of PLGA DTX ‐TIPS microparticle with precipitated DTX visible on the surface.

The amount of DTX loaded onto the TIPS microparticles was indirectly quantified by measuring the amount of DTX remaining in solution at different time points during the loading phase. Approximately 80% of the DTX was loaded onto the microparticles from the solution within 120 min of initiating mixing, matching the amount previously reported.^[^ ^14^ ^]^ Scanning electron microscopy of the microparticles confirmed the presence of crystalline DTX on the surface of the microparticles following incubation in the DTX solution, which was absent in unloaded control TIPS microparticles (**Figure** **1**).

### 2.2 Activity of DTX Released from the DTX‐TIPS Microparticles

**Figure 2:**
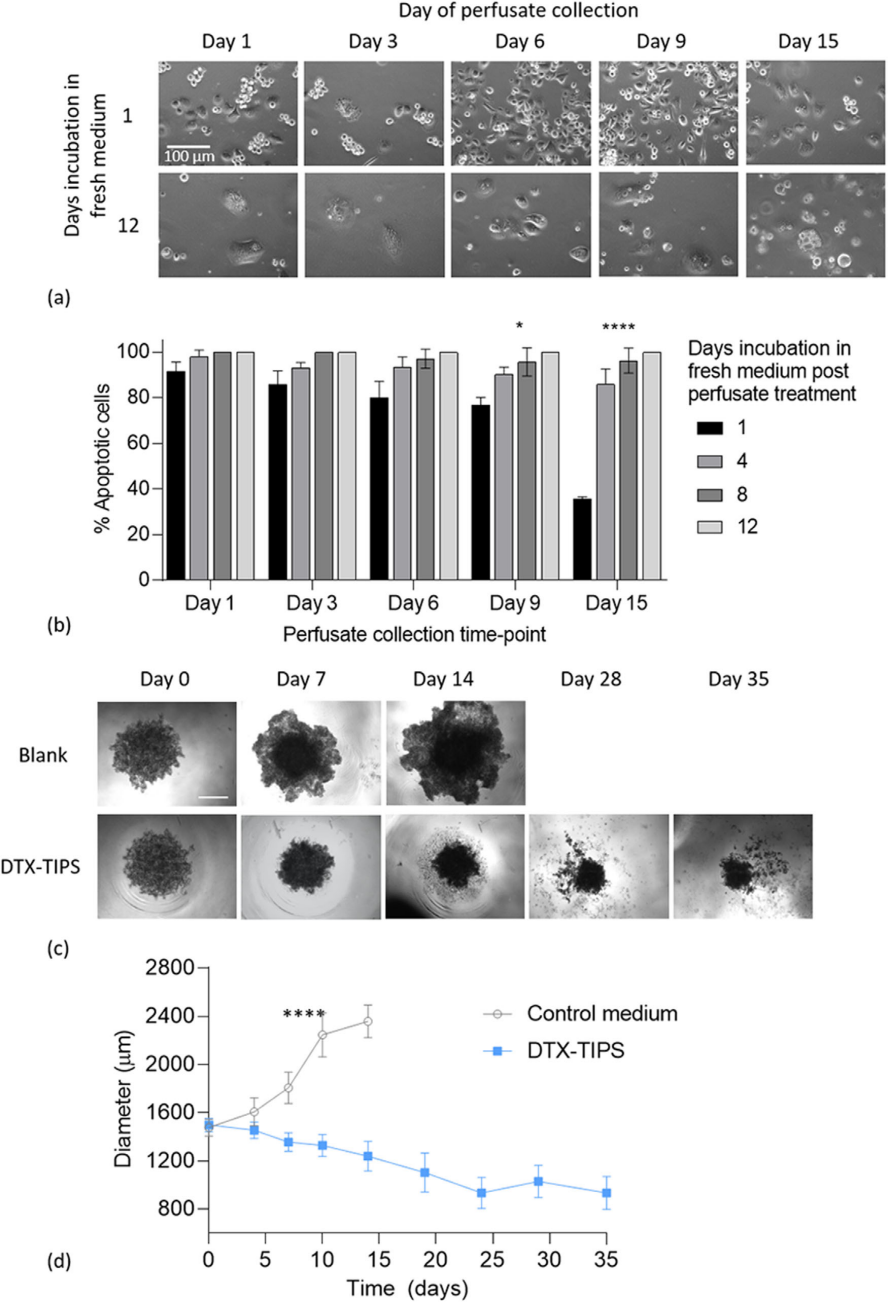
a) Phase contrast microscopy of PC3 prostate cancer cells incubated in DTX‐TIPS microparticle perfusate collected at days 1, 3, 6, 9, or 15 followed by incubation in fresh medium for 24 h (day 1) or 12 days. Cells at all time points are showing features of apoptosis resulting from exposure to DTX. b) Quantification of apoptotic PC3 cells in microscopy images of cells incubated in DTX‐TIPS perfusate collected at days 1, 3, 6, 9, or 15 followed by incubation in fresh medium for 1, 4, 8, or 12 days. Apoptosis is evident at all time points but the effects are less pronounced with perfusates collected at later time points after 1 day culture in fresh culture medium. Morphological features of apoptosis are present in 100% of cells for all perfusates at later time points following incubation in fresh culture medium. Data represent cells quantified from *n* = 2 fields of view per time point (*p < 0.05; ****p < 0.0001 versus perfusate collected at day 1). c) PC3 prostate cancer spheroids incubated with DTX‐TIPS perfusate collected at intervals to day 35 versus control group incubated in complete culture medium (scale bar: 200 µm). d) Change in PC3 prostate cancer spheroid size over 35 days (****p < 0.0001).

The in vitro activity of DTX released from TIPS microparticles was investigated in perfusate collected using a dynamic system. The perfusate containing DTX from TIPS microparticles was collected at regular intervals and evaluated for antineoplastic activity by assessing onset of apoptosis in cell‐based assays using the PC3 prostate cancer cell line. Phase contrast microscopy revealed characteristic morphological features of apoptosis including membrane blebbing, nuclear fragmentation, vesicular structure formation, and fragmentation into membrane‐bound apoptotic bodies (**Figure** **2**a). A sustained cytotoxic effect was observed in PC3 cells incubated in the DTX‐TIPS perfusate collected at different intervals over 15 days (Figure 2b). After 12 days incubation in fresh medium the cumulative toxic effects of DTX released into the perfusates collected at all time points resulted in fewer adherent cells remaining in the culture. The number of cells displaying apoptosis after 24 h incubation in perfusates collected at later time points was reduced when cultured in fresh medium at day 1 but increased to 100% after 12 days in fresh medium for all perfusates.

Further evaluation of the cytotoxicity of DTX in perfusates collected at time points beyond 15 days was conducted using 3D spheroid cultures composed of PC3 cells incubated with the perfusates collected over 35 days, with the culture medium being replenished at regular intervals corresponding to the time point of perfusate collection. The diameter of the spheroids incubated with medium conditioned with DTX‐TIPS microparticles, measured from images acquired as the experiment progressed, were significantly reduced in size compared with the diameter of the spheroids at corresponding time points in the control group incubated in nonconditioned complete medium (*p* < 0.0001; Figure 2c,d). The diameter of the spheroids in the treated group progressively decreased until day 24 before, followed by plateauing until the end of the experiment at day 35. In contrast, the diameter of spheroids in the control group incubated in nonconditioned complete medium continued to increase in size until at timepoints beyond 14 days their diameter exceeded the field of view so that measurements could no longer be collected for later timepoints.

**Figure 3:**
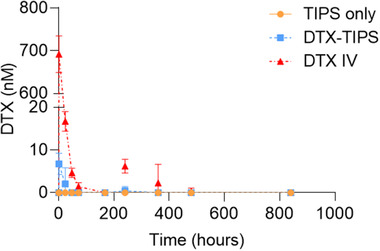
Measurement of DTX in plasma collected from nontumor bearing BALB/cAnNCrl mice (*n* = 5 per group). Low levels of DTX were detectable in plasma only at 1 and 24 h after delivery of DTX‐TIPS microparticles. Higher levels of DTX at 1 and 24 h were detected following I.V. DTX administration, with detectable levels of DTX remaining at 48 and 72 h and also at day 10 (240 h) and day 15 (360 h).

Analysis of the DTX release profile in vivo was investigated in plasma collected from nontumor bearing BALB/cAnNCrl mice. Detectable levels of DTX released from TIPS microparticles were observed at 1 and 24 h postadministration. DTX was not detectable in the plasma thereafter. Plasma samples collected from the group receiving I.V. DTX once weekly for three weeks contained significantly higher levels of DTX at 1 h (102‐fold increase) and 24 h (8‐fold increase) postadministration compared to the DTX‐TIPS group (*p* < 0.01) (**Figure** **3**). The administration of I.V. DTX resulted in detectable levels remaining in the plasma at 48 and 72 h and also at day 10 and day 15, corresponding to the second and third doses of I.V. DTX delivered at day 7 and day 14.

**Figure 4:**
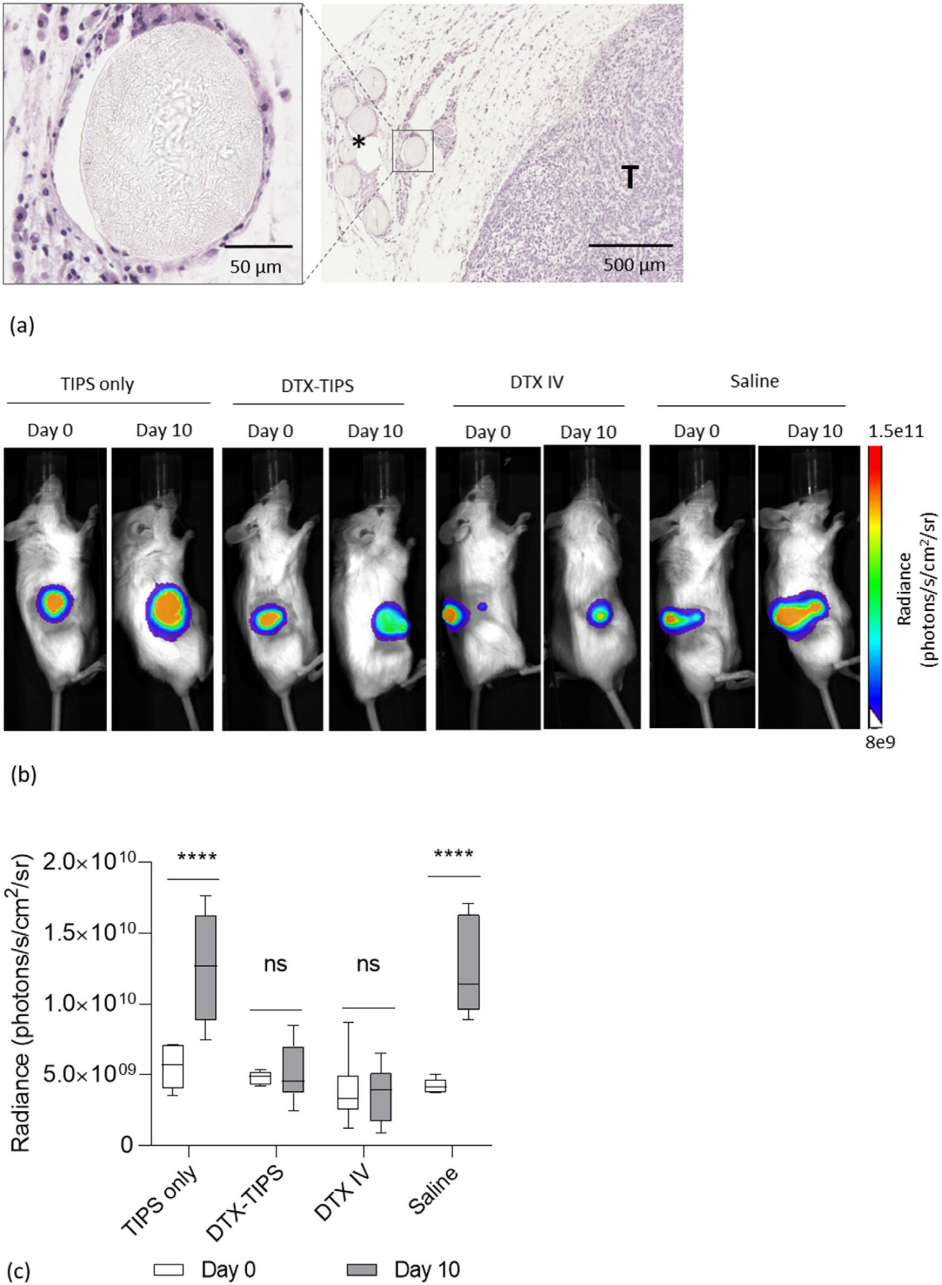
a) Histology of resected PC3 xenograft tumor (T). The DTX‐TIPS microparticles implanted peritumorally (*) remain at the site of delivery at day 35. The microparticles are surrounded by loose connective tissue. Higher magnification (inset) reveals the microparticles remain intact following implantation. b) Bioluminescence of luciferase‐positive PC3 prostate cancer xenografted tumors at day 0 versus day 10 for each treatment group. c) Bioluminescent signal in radiance (photons/s/cm^2^/sr) of the tumor at days 0 and 10 for TIPS only (*n* = 4), DTX‐TIPS (*n* = 4), DTX‐IV (*n* = 5), and saline groups (*n* = 3). Significantly increased bioluminescence (indicative of increased tumor volume) was observed between day 0 and day 10 for the groups receiving I.V. saline or TIPS only control microparticles (****p < 0.0001). No significant difference (ns) was observed between day 0 and day 10 for the groups receiving DTX‐TIPS or I.V. DTX.

The antineoplastic activity and systemic toxicity of DTX‐TIPS microparticles was investigated in vivo using a human prostate tumor xenograft model in NSG mice. PC3 cells were subcutaneously transplanted into immunocompromised mice. At day 14 postcell injection, palpable tumors had formed, measuring 0.03–0.05 cm^3^. Peritumoral delivery of TIPS microparticles (+/− DTX) and retention at the site of implantation was confirmed by histology, with the microparticles remaining in situ for the duration of the study (**Figure** **4**a). Following administration of the microparticles (+/− DTX) or I.V. DTX or saline, tumor growth was assessed over 33 days. Bioluminescence radiance, indicative of the tumor size, did not increase between day 0 and day 10 in the groups treated with I.V. DTX or DTX‐TIPS, whereas the signal was significantly increased in the groups receiving either saline of TIPS microparticles only (*p* < 0.0001) (Figure 4b,c).

**Figure 5:**
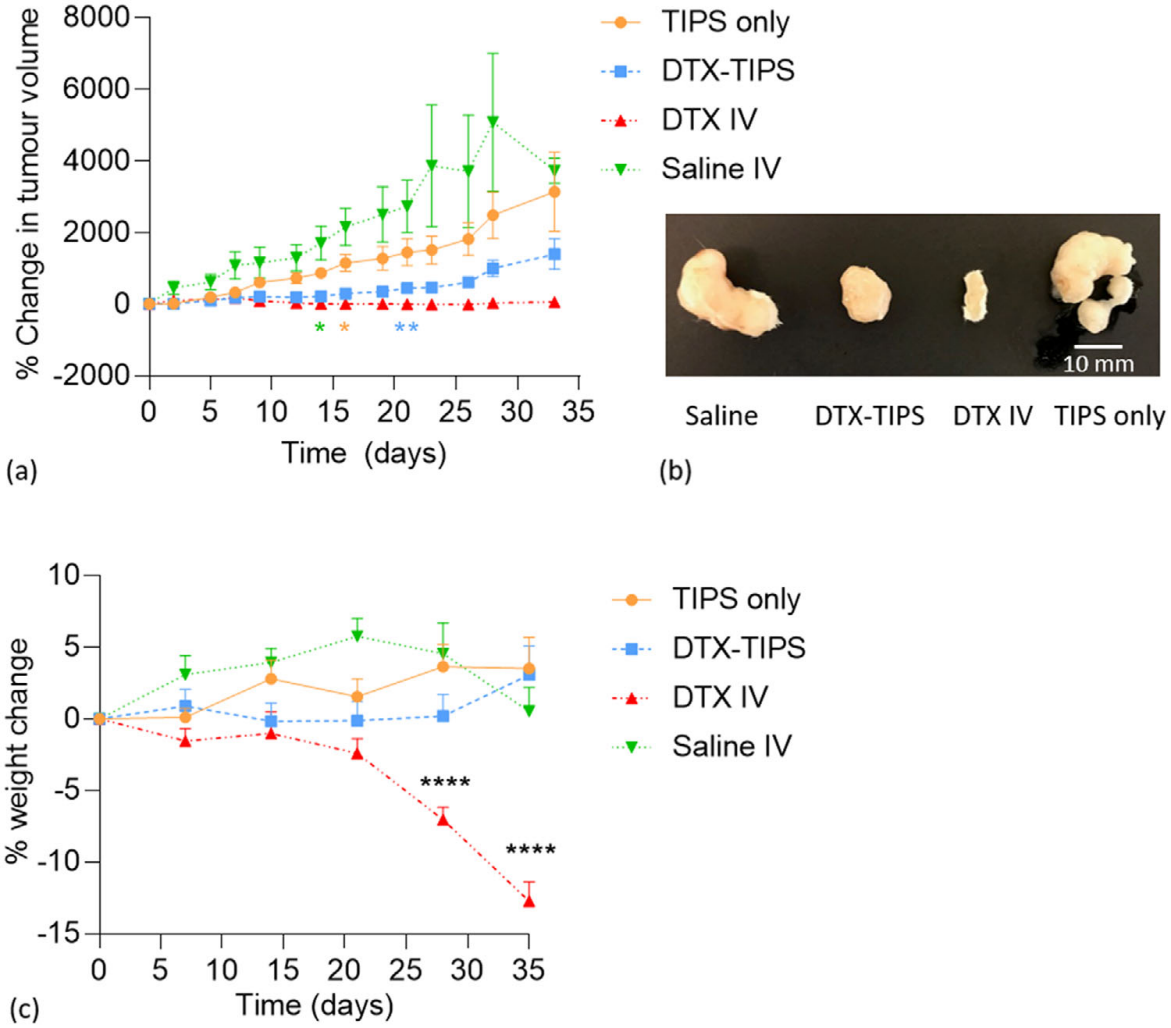
a) The greatest increase in tumor volume was observed in the groups receiving either I.V. saline or control TIPS microparticles. No increase in tumor volume was observed in the group receiving I.V. DTX. Tumor volume increase was attenuated in the group receiving DTX‐TIPS. (data values plotted are mean ± standard error of the mean. The *p* value color corresponds with treatment group versus DTX‐IV at indicated timepoint (*p < 0.05, **p < 0.01). (b) Macroscopic images of representative resected PC3 prostate cancer xenografted tumors illustrating the difference in tumor volume associated with the different treatment groups. c) Change in body weight of NSG immunodeficient mice following treatment. Progressive weight loss was observed in mice receiving I.V. DTX. No significant different weight change was observed in mice receiving DTX‐TIPS (data values plotted are mean ± standard error of the mean; ****p < 0.0001).

Tumor volume measurements in mice receiving I.V. DTX did not increase during the study (**Figure** **5**a). In mice receiving DTX‐TIPS microparticles, there was no significant increase in tumor volume compared with the mice receiving I.V. DTX until day 21 (*p* < 0.01). At this time point, the increase in tumor volumes in the group treated with DTX‐TIPS microparticles was approximately sixfold lower compared with the group treated with saline only at the same time point. In mice receiving either TIPS microparticles only or I.V. saline, the tumor volume was significantly increased at day 16 (*p* < 0.05) and day 14 (*p* < 0.05), respectively, compared with the mice receiving I.V. DTX. Qualitative macroscopic assessment of the resected tumors at the end of the study revealed visibly smaller tumors collected from the groups treated with DTX (Figure 5b).

Significant toxicity was associated I.V. delivery of DTX, exhibited by progressive weight loss from day 7 onward, leading to −7.0% ± 2.3% at day 28 and −12.7% ± 3.5% at day 35 compared with the starting weight (*p* < 0.0001; weight loss exceeded >15% at day 35 in 3/7 mice). In contrast, no significant weight loss was observed in the groups receiving DTX‐TIPS microparticles, TIPS microparticles only or I.V. saline (Figure 5c).

Tissue levels of DTX in explanted organs (heart, kidney, lung, spleen, and liver) were analyzed days 1, 10, and 35 postadministration (**Table** **1**). Elevated levels of DTX were detected in all organs at all time points following I.V. administration of DTX compared with DTX‐TIPS administration, with the exception of the liver and spleen at day 35, where DTX was not detectable for either treatment group.

**Table 1:** DTX concentration measured in tissues from explanted organs at days 1, 10, and 35 following administration of DTX‐TIPS microparticles or I.V. DTX (*n* = 5–6 samples for each group per time point)

	DTX concentration (×10^−9^ m)Days postadministration					
	1		10		35	
	DTX‐TIPS	I.V.	DTX‐TIPS	I.V.	DTX‐TIPS	I.V.
Heart	11.25 ± 6.50	339.87 ± 42.88	0.00 ± 0.00	90.08 ± 19.43	0.00 ± 0.00	0.97 ± 2.37
Kidney	18.93 ± 8.94	114.78 ± 22.36	5.28 ± 7.74	114.78 ± 22.36	0.00 ± 0.00	1.167 ± 2.86
Lung	0.00 ± 0.00	641.02 ± 92.42	4.28 ± 5.94	256.08 ± 35.54	0.00 ± 0.00	13.75 ± 16.20
Liver	10.05 ± 5.85	87.30 ± 7.85	16.22 ± 22.84	27.04 ± 11.88	0.00 ± 0.00	0.00 ± 0.00
Spleen	0.00 ± 0.00	14.07 ± 11.86	0.00 ± 0.00	35.28 ± 51.89	0.00 ± 0.00	0.00 ± 0.00

Clinical utility of taxane‐based drug formulations in cancer is often limited by chemotherapy dose‐limiting toxicity, nonresponders and resistance.^[^ ^18^ ^]^ Conventional modes of delivery for many chemotherapeutics involve intravenous administration of multiple doses, resulting in much of the drug not reaching the diseased tissue. New approaches for drug delivery that involve delivery directly to the target site would alter the pharmacokinetic profile and biodistribution of the chemotherapeutic agent and improve its therapeutic index. This approach would be beneficial for high‐risk patients with localized disease where there is a higher likelihood of a positive resection margin following surgical extirpation.^[^ ^9^ ^]^

To mitigate the risk of residual cancer cells remaining due to positive surgical margins following radical prostatectomy, delivery of therapeutic quantities of a chemotherapeutic agent, such as DTX, into the postsurgical tissue cavity would directly target these residual tumor cells and potentially reduce disease recurrence whilst avoiding systemic toxicity associated with systemic administration. Furthermore, increasing the bioavailability of DTX at the tumor site may also reduce development of resistance.^[^ ^19^ ^]^

We envisage delivery of highly porous biodegradable TIPS microparticles coated with DTX into the postsurgical tissue cavity following radical prostatectomy in all patients could provide an effective prophylactic measure to mitigate the risk of residual tumor cells that may remain and thus diminish the risk of disease recurrence. For this approach to be clinically adopted it will require evidence that the DTX released is effective against the tumor cells but does not cause systemic toxicity associated with I.V. delivery. The current study explored this concept further and demonstrated that DTX‐TIPS microparticles can deliver sustained amounts of DTX targeted to tumors that is effective in attenuating tumor growth without causing systemic toxicity. The level of tumor growth inhibition achieved following peritumoral delivery of a single dose of DTX‐TIPS microparticles was similar to that achieved following I.V. delivery of three doses of DTX at weekly intervals. Intravenous DTX for the treatment of metastatic prostate cancer is often given over six cycles.

The antineoplastic (and cytotoxic) activity of DTX is well established and involves binding to intracellular β‐tublin and activation of apoptosis.^[^ ^10^ ^]^ This activity was confirmed in vitro using 2D and 3D model systems, which exhibited signs of apoptosis and cytotoxicity similar to reported in our previous study.^[^ ^14^ ^]^ The findings from the current in vitro study demonstrated that the antineoplastic effect of DTX released from TIPS microparticles was sustained and lasted at least up to 35 days. Interestingly, the potency of the cytotoxic effect observed in the 2D model after 1 day incubation in fresh medium postperfusate treatment became more diminished at later perfusate collection time points (indicated by fewer cells displaying features of apoptosis). However, further incubation of the treated cells in fresh medium up to 12 days coincided with all of the cells displaying features of apoptosis. This suggests that although the amount of DTX released from DTX‐TIPS was reduced at later time points, the concentration was still sufficient to activate apoptotic pathways.

The sustained activity of DTX released from TIPS microparticles in vitro corresponded with the tumor growth inhibitory effect observed in vivo. DTX‐TIPS microparticles attenuated significant tumor growth up to day 21 postadministration. It is worth noting that at day 21, the group receiving I.V. DTX had received three doses of DTX (the most recent being at day 14), which would account for the continued inhibition of tumor growth in this group beyond day 21. The I.V. dose of DTX chosen for the current study was equivalent to the dose previously reported for murine xenografted tumor growth inhibition studies.^[^ ^20^, ^21^ ^]^ The I.V. formulation consisted of DTX dissolved in ethanol together with the nonionic surfactant excipient Polysorbate 80, producing a formulation that is similar to the clinically approved injection formulations of DTX (e.g., Taxotere) that also contain Polysorbate 80 to stabilize aqueous formulations for parenteral administration. Although mice in the I.V. DTX group displayed sustained inhibition of tumor growth, at time points beyond day 21 all mice in this group showed signs of progressive toxicity, demonstrated by significant weight loss at days 28 and 35. In contrast, mice that received DTX‐TIPS did not exhibit weight loss at the later time points, while still displaying evidence of tumor inhibition compared the control groups receiving TIPS microparticles only or I.V. saline. Systemic toxicity following the I.V. dosing regimen corresponded with increased levels of DTX present in the plasma and organ tissues over an extended period of time, which was absent in the DTX‐TIPS microparticle treated group. The detection of DTX in organ tissues but not in plasma at day 35 following I.V. delivery was a surprising observation. A possible explanation for this is binding of DTX to plasma proteins which would prevent it from being detected by liquid chromatography–mass spectrometry analysis without further sample extraction procedures. This effect could also account for the level of DTX in the plasma at 24 h being <5% of the quantity detected at 1 h postadministration

Recent years have seen a shift toward the development of various taxane‐based nanoformulations (liposome, micelle, nanoparticle, and nanoemulsion), with many incorporating advanced functionality to assist drug binding to specific sites following I.V. delivery. However, challenges with nanotechnology include quality control, physicochemical stability, storage conditions, scale‐up manufacture, in vivo metabolism and off‐target migration.^[^ ^22^ ^]^ The current proof‐of‐concept study demonstrates the feasibility of using microparticles for targeted delivery of toxic chemotherapeutic agents. Such microscale delivery systems offer several advantages when targeting positive surgical margins. The study demonstrates that the microparticles are small enough for delivery into the tissue, for example though a needle or cannula, whilst being large enough to prevent migration from the site of delivery. The latter feature is an important attribute for ensuring the drug release remains localized. The composition of PLGA TIPS microparticles used in the current study has been scaled up for investigation in other clinical conditions. These studies have demonstrated that TIPS microparticles have long‐term stability if stored under dry conditions (6 years plus; data not shown). Findings from the current study show that DTX‐TIPS microparticles are compatible with dry storage but will require further investigation to establish the long‐term stability of the combined formulation.

A variety of acute and long‐term side effects are associated with commercial formulations of DTX. These include infusion reactions, febrile neutropaenia, fatigue, pneumonitis, and peripheral neuropathy.^[^ ^13^ ^]^ To facilitate DTX loading of TIPS microparticles via antisolvent precipitation the formulation of DTX used in the current study omitted the inclusion of Polysorbate 80, which has been implicated in causing systemic and injection site adverse events.^[^ ^23^ ^]^

Efficacious clinical use would require the DTX‐TIPS microparticles to release an adequate amount of DTX to destroy all remaining tumor cells in the prostate bed. Further refinement of the DTX‐TIPS microparticles to achieve the necessary antineoplastic potency might be achieved by loading a greater quantity of DTX onto the surface of the microparticles. This could deliver a higher payload of DTX to the diseased tissue but care would need to be taken to avoid elevated concentrations entering the systemic circulation causing toxicity. Our previous study showed that ≈95% of DTX is released from the current composition of DTX‐TIPS microparticles during the first 5 days, with approximately one third being released during the first 24 h.^[^ ^14^ ^]^ An alternative approach for achieving enhanced potency would be to alter the composition of the TIPS microparticles to a polymer that degrades more slowly and releases DTX over a longer period. However, histology at the end of the current study indicated that TIPS microparticles were largely intact and retained in the peritumoral location of delivery, indicating higher drug loading rather than slower degradation would be beneficial.

The DTX‐TIPS microparticles represent an integral drug‐device combination product, where the primary mode of action of the product is pharmacological, through the antineoplastic action of DTX. In addition to the action of the DTX, it is likely that the TIPS microparticle component of the product will also provide a temporary tissue scaffold that might also facilitate tissue healing after prostate removal. Since the current study demonstrated persistence of TIPS microparticles at a heterotopic site further investigation will be required to evaluate the likely safety and benefit of the microparticle device component of the product following delivery after prostate gland removal during radical prostatectomy surgery. TIPS microparticles alone have been approved for clinical testing for use as investigational Class III medical device. As part of their development process, we have confirmed compliance with ISO 10993 (biocompatibility), with data demonstrating a lack of antigenicity and minimal tissue reaction.

If DTX‐TIPS microparticles are to be delivered into the prostate cavity at the time of surgery for all patients undergoing radical prostatectomy early phase clinical safety testing will be necessary to demonstrate avoidance of immediate toxicity, bleeding, infection, anastomotic leak, and impediment of healing.

A caveat of the experimental approach used in the current study is the peritumoral delivery of DTX‐TIPS microparticles around intact tumors in the xenograft model instead of the proposed clinical approach consisting of delivery into a tissue cavity containing residual tumor cells that exists in patients with positive resection margins following tumor excision. The size and location of the xenografted tumors made it impossible to simulate the presence of residual cancer cells in a tissue cavity following tumor resection. It is possible, therefore, that the model used to demonstrate the antineoplastic activity of DTX‐TIPS microparticles may reflect an over representation of what is required for the intended clinical use of targeting fewer residual tumor cells following radical prostatectomy. Furthermore, previous studies have shown extensive binding of DTX to plasma proteins, including α1‐acid glycoprotein, albumin and lipoproteins.^[^ ^24^ ^]^ Therefore, the therapeutic index of DTX released from the microparticles into an environment immediately following surgery may also be influenced by the milieu of tissue fluid and plasma proteins, as well as the inflammatory response arising from tissue trauma associated with surgical extirpation.

The findings from the current study indicate DTX‐TIPS microparticles could lead to improved patient outcomes if similar DTX concentrations can be achieved in the prostate bed with limited distribution to plasma and normal tissue. Localized, sustained delivery of the chemotherapeutic agent using the approach reported would avoid the need for pelvic radiotherapy and genitourinary side effects, and reduce the amount of healthcare time and costs, as well as patient inconvenience, associated with follow‐up clinical visits. Early phase clinical testing will be needed in a small group of patients to enable evaluation of safety of DTX‐TIPS microparticles delivered during surgery for high‐risk patients undergoing radical prostatectomy (e.g., reduced incidence of severe (CTCAE G3+) treatment‐related toxicity relative to historical control rates).

## 3 Conclusion

Collectively, the results from the current study indicate DTX‐TIPS microparticles provide a safe and efficacious approach for delivering highly toxic chemotherapeutic agents whilst avoiding systemic toxicity. The combination product offers a new approach to adjuvant chemotherapy that is facile in execution for targeting residual tumor cells in situ resulting from a positive resection margin. This might allow patients to receive clinically transformative doses of DTX without toxicity and side‐effects of conventional systemically administered chemotherapy. Although the current study has focused on use in prostate cancer, utility of DTX‐TIPS microparticles poses minimal barriers to clinical adoption. Furthermore, its use may extend beyond prostate cancer and include patients undergoing surgical resection of other solid cancers where there is a risk of positive surgical margins or in anatomical locations where there is a need to minimize the extent of surgery to preserve tissue function.

## 4 Experimental Section

### Fabrication of TIPS Microparticles

TIPS microparticles composed of PLGA were prepared as previously described.^[^ ^14^ ^]^ PLGA PURASORB 7507 (75:25) polymer (Corbion, Amsterdam, Netherlands) was dissolved in dimethyl carbonate (Sigma Aldrich, Dorset, UK) overnight using magnetic stirring to produce a 10% (w/v) polymer solution. The polymer solution then was fed into a Nisco Encapsulator Unit (Nisco Engineering, Zurich, Switzerland; Frequency: 2.75 kHz, Amplitude: 70%) by a syringe pump (Harvard Apparatus, Kent, UK), at a constant flow rate of 2 mL min^−1^. The polymer droplets were formed using a 100 µm sapphire nozzle and collected in liquid nitrogen. Residual solvent was removed from the frozen polymer droplets by lyophilization for 48 h. The dried PLGA TIPS microparticles were sieved to a size range of 250–350 µm and stored at room temperature in rubber stoppered glass vials under vacuum.

### Loading TIPS Microparticles with DTX

DTX (Cayman Chemical, USA) was loaded onto TIPS microparticles using an antisolvent precipitation method.^[^ ^14^ ^]^ 5 mg of PLGA TIPS microparticles was transferred into 20 mL clear type 1B borosilicate glass vials and sealed with a butyl injection stopper. 4.5 mL of ultrapure water was added to the vial and vortexed for 10 s. 0.5 mL of 1 mg mL^−1^ DTX in ethanol was added using 1 mL syringe with a 25 G needle through the rubber stopper. The vial was then vortexed for 10 s and placed on the roller mixer (IKA Roller 6 digital; 60 rpm) at room temperature for 2 h.

DTX loading efficiency (DLE) onto the TIPS microparticles after 2 h incubation was calculated according to the following equation. The amount of free DTX left in the solution was measured by UV spectroscopy at the wavelength of 229 nm using a Nanodrop 2000c spectrophotometer (Thermo Scientific, Waltham, MA).

Unbound DTX and ethanol solution was removed from the microparticles by washing 3 × 5 mL ultrapure water, followed by desiccation under vacuum. Samples of the dried microparticles were coated with gold for 60 s using a Q150R ES gold coater (Quorum Technologies, Oxford, UK). Scanning electron microscopy (SEM; Hitachi S3400N scanning electron microscope) was used to confirm the presence of DTX on the surface of TIPS microparticles.

### In Vitro Activity of DTX Released from the DTX‐TIPS Microparticles—In Vitro Collection of DTX‐TIPS Microparticle Conditioned Perfusate

Sustained release of DTX from the TIPS microparticles in vitro and its biological activity was investigated using a dynamic perfusion system to simulate release in a physiological environment when delivered following radical prostatectomy. DTX‐loaded microparticles were mixed with 100 µL of 70% (v/v) GranuGel (Convatec, UK) diluted in ultrapure water and the mixture was placed between two 25 mm circular filter papers (Whatman qualitative cellulose filter paper, Grade 1), where their positions were held by a Swin‐Lok plastic membrane filter holder. A hypodermic needle (18G × 40 mm) connected to the outlet of the filter holder, was inserted through the lid of a 50 mL polypropylene container to collect the perfusate. The perfusion system was placed inside an incubator at 37 °C and complete culture medium (Ham's F12‐K medium (Kaighn's modification) (Invitrogen) supplemented with 10% (v/v) fetal bovine serum (FBS) and 1% antibiotics) was used as the perfusate to simulate physiological tissue fluid. The perfusate was pumped through the filter holder using a peristaltic pump (Harvard Apparatus) at a flow rate of 0.01 mL min^−1^. Conditioned perfusate was sampled at specified intervals and used to evaluate its biological activity in vitro.

### In Vitro Activity of DTX Released from the DTX‐TIPS Microparticles—In Vitro Biological Activity of DTX‐TIPS Microparticle Conditioned Perfusate

Human prostate cancer cells (PC3, American Type Culture Collection) were used to test the activity of DTX released from the DTX‐TIPS microparticles. PC3 cells were maintained in complete culture medium. Cells were cultured at 37 °C under 5% CO_2_ atmosphere in a humidified incubator.

To assess the longevity of biologically active DTX released from TIPS microparticles, morphological changes indicative of apoptosis (including membrane blebbing, nuclear fragmentation, vesicular structure formation, and fragmentation into membrane‐bound apoptotic bodies) were quantified in PC3 cells maintained in 2D culture following incubation for 24 h in the perfusate. The conditioned complete medium was collected over a 24 h period at different perfusion time points (days 1, 4, 8, and 12) and added to PC3 cells seeded in six‐well plates at 1.5 × 10^5^ per well. After 24 h incubation, the medium was replaced with fresh complete medium, which was replenished every 48 h for up to 15 days. Images of cell morphology were acquired using phase contrast microscopy using a Zeiss Primovert microscope and at least 25 cells in each group were analyzed to calculate the number of cells displaying apoptosis.

3D spheroids of PC3 cells were generated using methylcellulose as a scaffold, as previously described.^[^ ^25^ ^]^ PC3 were seeded at a concentration of 2 × 10^4^ cells/200 µL complete culture medium containing 20 wt% methylcellulose in 96‐well ultralow attachment u‐bottom plates. The cells were incubated for 2 days at 37 °C under 5% CO_2_ atmosphere in a humidified incubator until the spheroids had formed. The medium was replaced with 200 µL perfusate conditioned complete medium that was replaced with medium collected from the perfusate system at the corresponding time point. Images of the spheroids were acquired for each day of the culture. The dimensions of the imaged spheroids were measured using Image J. Feret's diameter was used to estimate the mean diameter of spheroids and plotted against time (GraphPad Prism Version 8.0; GraphPad Software, San Diego, USA).

### In Vitro Activity of DTX Released from the DTX‐TIPS Microparticles—Measurement of DTX Released In Vivo from the DTX‐TIPS Microparticles

To determine the concentrations of DTX present in the circulation post‐treatment, DTX‐TIPS microparticles were implanted subcutaneously into nontumor bearing BALB/cAnNCrl mice (*n* = 5) (7–8 weeks, 17–20 g, Charles River). Control animals received either control TIPS microparticles (5 mg TIPS microparticles after mixing into a suspension in 100 µL of 70% (v/v) GranuGel; *n* = 5) or I.V. DTX (10 mg kg^−1^ via tail vein delivery once weekly for three weeks (*n* = 5). (The I.V. formulation of DTX was prepared by dissolving 10 mg DTX in 500 µL of absolute ethanol. Once fully dissolved, 500 µL of Polysorbate 80 was added and gently mixed to obtain a presolution that was finally diluted 1:10 (v/v) in saline to produce 1 mg mL^−1^ DTX.) For measurement of circulating DTX in plasma, blood samples were collected via the tail vein at day 0 (predose), and days 1, 2, 3, 7, and 10 then every 5 days until day 35 immediately before the end of in life phase. Blood samples were collected into 10 µL capillaries containing sodium heparin (Hirschmann, Eberstadt, Germany) and placed in wells of a 2 mL deep well plate that was stored at −80 °C until extraction.

On the day of the extraction, calibration curve and sample plates were thawed. 125 µL of 70:30 water:acetonitrile containing 0.4% blood was added to blanks, calibration curve and QCs. 125 µL of 70:30 water:acetonitrile containing 0.4% DMSO was added to all samples. 40 µL of each blood:water:acetonitrile sample was taken and protein precipitated with 120 µL acetonitrile containing DTX‐d_9_ (25 × 10^−9^ m; Cayman Chemicals, USA, item 222094). Samples were mixed and centrifuged. Supernatant was taken and diluted 40:60 with 0.5% sodium acetate (20 × 10^−6^ m) in ammonium acetate (10 × 10^−3^ m, pH 5).

Liquid chromatography–mass spectrometry analysis was carried out with a Waters (Milford, MA) H‐class Acquity solvent manager and sample manager on a Waters Acquity HSS PFP column (1.8 µm, 50 mm × 2.1 mm id) with a gradient consisting of 10 × 10^−3^ m ammonium acetate (pH 5.0) and acetonitrile mobile phases. The flow rate was 0.6 mL min^−1^ and the run time 5.6 min. Analyte and internal standard were ionized using electrospray ionization in positive ion mode. Detection of analytes was via tandem mass spectrometry (MS/MS) using a Waters Xevo TQ‐S mass spectrometer in the multiple reaction monitoring (MRM) mode. For DTX and DTX‐d_9_ (IS), the transitions *m*/*z* 830.4–248.1/304.2 and *m*/*z* 839.4–313.0 were monitored, respectively. The calibration curve was linear over the concentration range (1–10 000) × 10^−9^ m.

### In Vitro Activity of DTX Released from the DTX‐TIPS Microparticles—In Vivo Tumor Growth Inhibitory Activity

Female NSG immunodeficient mice (NOD.Cg‐PrkdcscidIl2rgtm1Wjl/SzJ; Charles River), aged 6–7 weeks and 18–20 g in body weight, were acclimatized for 1 week prior to injection of tumor cells. PC3 cells (5.0 × 10^6^ cells in 100 µL phosphate buffered saline (PBS)) were injected subcutaneously into the right flank of each mouse. The dimensions of tumor were measured three times per week using digital calipers and the tumor volume was calculated using the following formula

(1) $$\mathrm{Tumor}\;\mathrm{volume}\left(cm^{3}\right)=\left(W/10\times L/10\times H/10\right)\times \pi /6$$

At day 14 postcell injection, the mice were randomized into four groups: Group 1 intravenous (I.V.) DTX (10 mg kg^−1^ via tail vein delivery once weekly for three weeks; *n* = 7); Group 2 peritumoral injection of DTX‐TIPS microparticles (5 mg of TIPS microparticles loaded with 500 µg DTX; *n* = 7); Group 3 peritumoral injection of control TIPS microparticles (5 mg of control TIPS microparticles; *n* = 7); Group 4 I.V. saline (10 µL g^−1^ body weight; *n* = 4). Mice in Groups 2 and 3 received TIPS microparticles (+/− DTX) after mixing the microparticles into a uniform suspension in 100 µL of 70% (v/v) GranuGel. The suspension of TIPS microparticles in GranuGel was delivered subcutaneously via 1 × 100 µL depot using a 1 mL syringe and 16G needle around the periphery of the tumor. Mice were monitored for signs of toxicity (weight loss, body condition, and uncoordinated movement) over the duration of the study. Mice were euthanized if weight loss exceeded 15% of the starting body weight or they showed excessive signs of toxicity.

At the end of the in‐life phase (days 1, 10, and 35 post‐treatment) the mice were euthanized by overdose of CO_2_, followed by cervical dislocation and organs (heart, liver, kidneys, lungs, and spleen) were collected, weighed and immediately frozen in liquid nitrogen before storage at −80 °C until further analysis. Evaluation of DTX concentrations was carried out by liquid chromatography tandem mass spectrometry in tissue following homogenization in 3 mL g^−1^ (spleens 5 mL g^−1^) 10 × 10^−3^ m PBS using a Precellys 24 homogenizer (Bertin Technologies, Montigny‐le‐Bretonneux, France). 45 µL tissue homogenate was spiked with 5 µL DMSO, samples were mixed and protein precipitated with 150 µL acetonitrile containing DTX‐d_9_ as internal standard (25 × 10^−9^ m). Samples were mixed and centrifuged. Supernatant was taken and diluted 40:60 with 0.5% sodium acetate (20 × 10^−6^ m) in ammonium acetate (10 × 10^−3^ m, pH 5). Blanks, calibration curve and QCs were prepared as above using tissues obtained from the same strain of mice (NSG) and spiked with DMSO, working calibration standard and working QC, respectively. Blanks consisted of protein precipitated with acetonitrile alone, blank+, standards, and QCs were protein precipitated with acetonitrile containing DTX‐d_9_.

The tumors were explanted and processed for histology. Tissues were fixed in 10% formalin, dehydrated and embedded in low‐melting point wax (Paraplast X‐TRA, Sigma). Tissue sections cut from the wax‐embedded tissue were stained with hematoxylin and eosin.

### Generation of Luc‐GFP Expressing PC3 Cell Line

Lentivirus encoding firefly luciferase and GFP was produced in HEK 293 T cells using a calcium phosphate precipitation protocol adapted from that described by Tiscornia et al.,^[^ ^26^ ^]^ using the transfer plasmid pSEW‐Flagx3‐FLuc‐2A‐GFP (which was a kind gift from Dr. Martin Pule, UCL Cancer Institute), together with packaging plasmids, Gag‐pol (pCMV‐R8.74; Addgene Plasmid # 22036) and VSV‐G (pMD2.G; Addgene Plasmid # 12259). To improve viral titers, sodium butyrate (1 × 10^−3^ m) was added to the media, 24 h prior to lentiviral harvest.^[^ ^27^ ^]^ Lentivirus was harvested into PC3 culture medium, passed through a 20 µm syringe filter, and added directly to PC3 cells growing at 40% confluence for transduction. After 24 h PC3 cells were changed into fresh media, and successful transduction noted after a further 24 h with over 90% +ve GFP expression seen using fluorescence microscopy.

### Whole Body Bioluminescence Imaging

For bioluminescence imaging (BLI) of the tumor, PC3 cells were transduced with recombinant lentivirus to express the luciferase gene, as described above. Luciferase ± PC3 cells (5 × 10^6^ in 100 µL PBS) were injected subcutaneously into the right flank of female NSG immunodeficient mice. At day 14 postcell injection, the mice were randomized into four groups: Group 1 intravenous (I.V.) DTX (10 mg kg^−1^ via tail vein delivered once; *n* = 5); Group 2 peritumoral injection of DTX‐TIPS microparticles (*n* = 4); Group 3 peritumoral injection of control TIPS microparticles (*n* = 4); Group 4 I.V. saline (10 µL g^−1^ body weight; *n* = 3). BLI was performed using IVIS Lumina (PerkinElmer USA). Mice were injected intraperitoneally with 75 mg kg^−1^ d‐luciferin (Promega) in 200 µL of PBS. Sequential BLI images were acquired 5 min after luciferin injection using 1 s exposure time with 1 min delay between two consecutive acquisitions. BLI images were analyzed using Aura Imaging Software (Spectral Imaging, USA) where a free draw region of interest (ROI) around the whole body and control circular background ROI was placed on the first image and subsequently pasted over each new image acquired until all ROIs reached their maximum intensity. The photon signal in the maximal signal ROI was quantified as Radiance (photons/s/cm^2^/sr). Representative images underwent thresholding to the same scale and presented using radiance (photons/s/cm^2^/sr) as color scale by utilizing the same software.

All in vivo experiments were performed under a UK Home Office license (PLN: 70/8421), in compliance with the 1986 United Kingdom Home Office Animals (Scientific Procedures) Act and with the approval of the University College London local ethics committee.

### Statistical Analysis

Data were tested for statistical significance using GraphPad Prism software (version 8.0; GraphPad Software San Diego, CA), with sample size (*n*) and values indicated in the figure legends. Statistical evaluation of differences between groups was performed by two‐way analysis of variance (ANOVA) with Sidak's multiple comparisons test with a single pooled variance used to compare differences between two or more groups. Data values are plotted as mean ± standard deviation (SD) unless stated otherwise in the figure legend.

## Conflict of Interest

A patent application has been submitted by UCL Business covering the combination of DTX and TIPS microspheres.
